# Impacts from Partial Removal of Decommissioned Oil and Gas Platforms on Fish Biomass and Production on the Remaining Platform Structure and Surrounding Shell Mounds

**DOI:** 10.1371/journal.pone.0135812

**Published:** 2015-09-02

**Authors:** Jeremy T. Claisse, Daniel J. Pondella, Milton Love, Laurel A. Zahn, Chelsea M. Williams, Ann S. Bull

**Affiliations:** 1 Vantuna Research Group, Department of Biology, Occidental College, Los Angeles, California, United States of America; 2 Marine Science Institute, University of California Santa Barbara, Santa Barbara, California, United States of America; 3 Pacific Region, Environmental Sciences Section, Bureau of Ocean Energy Management, Camarillo, California, United States of America; Northwest Fisheries Science Center, NOAA Fisheries, UNITED STATES

## Abstract

When oil and gas platforms become obsolete they go through a decommissioning process. This may include partial removal (from the surface to 26 m depth) or complete removal of the platform structure. While complete removal would likely eliminate most of the existing fish biomass and associated secondary production, we find that the potential impacts of partial removal would likely be limited on all but one platform off the coast of California. On average 80% of fish biomass and 86% of secondary fish production would be retained after partial removal, with above 90% retention expected for both metrics on many platforms. Partial removal would likely result in the loss of fish biomass and production for species typically found residing in the shallow portions of the platform structure. However, these fishes generally represent a small proportion of the fishes associated with these platforms. More characteristic of platform fauna are the primarily deeper-dwelling rockfishes (genus *Sebastes*). “Shell mounds” are biogenic reefs that surround some of these platforms resulting from an accumulation of mollusk shells that have fallen from the shallow areas of the platforms mostly above the depth of partial removal. We found that shell mounds are moderately productive fish habitats, similar to or greater than natural rocky reefs in the region at comparable depths. The complexity and areal extent of these biogenic habitats, and the associated fish biomass and production, will likely be reduced after either partial or complete platform removal. Habitat augmentation by placing the partially removed platform superstructure or some other additional habitat enrichment material (e.g., rock boulders) on the seafloor adjacent to the base of partially removed platforms provides additional options to enhance fish production, potentially mitigating reductions in shell mound habitat.

## Introduction

**Fig 1:**
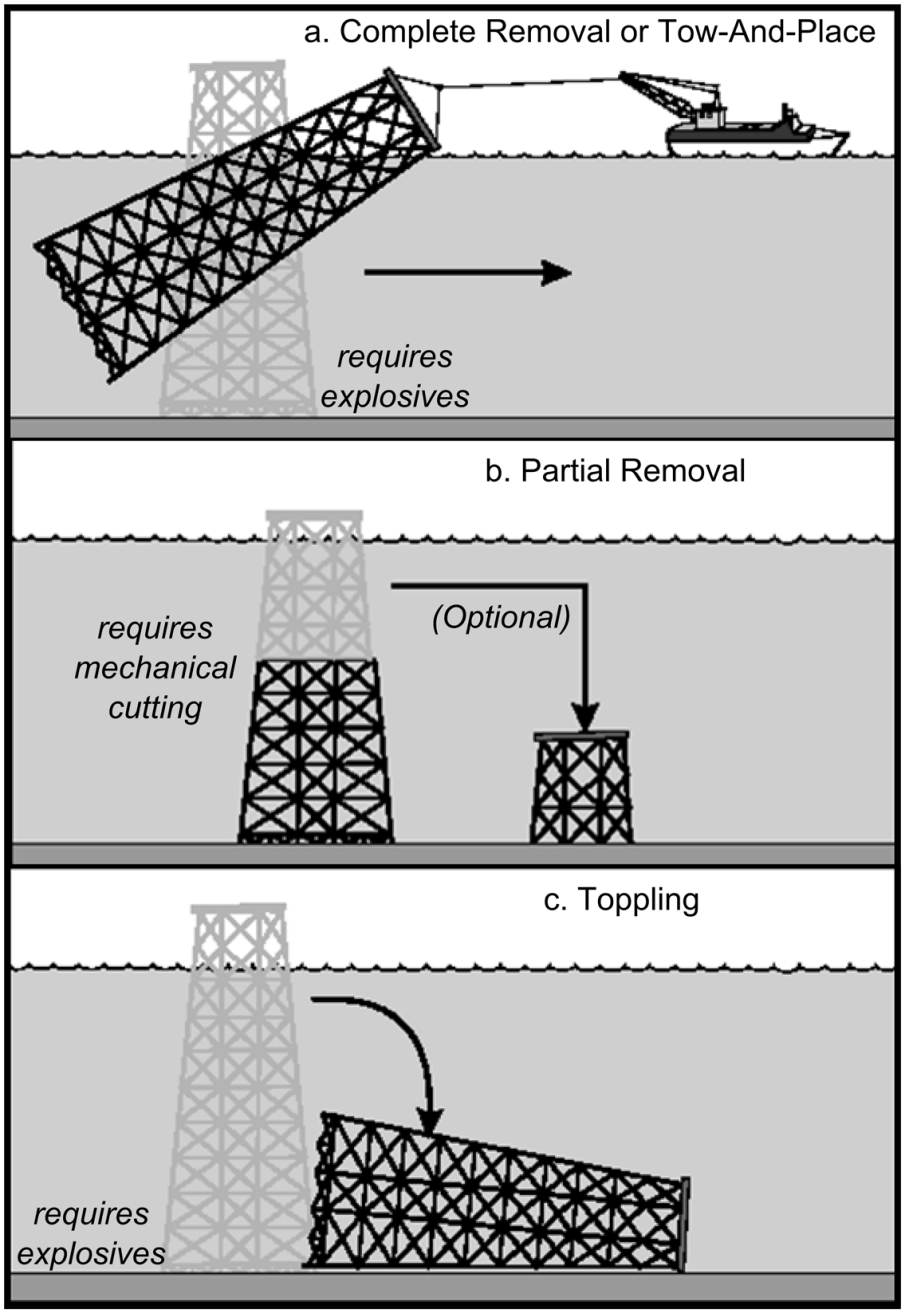
Reefing options for decommissioned oil and gas platforms. After all wells are permanently sealed, decommissioning may encompass one of four alternatives for the platform [2–4]: (a. Complete Removal) explosives are detonated to sever the well conductors, pilings, and support legs 5 m below the seafloor and the structure is towed to shore and scrapped, (a. Tow-And-Place) the severed structure is towed to a designated reef location and placed on the seafloor, (b. Partial Removal) the well conductors, pilings, and support legs are mechanically cut off, often at 26 m depth, and then optionally placed back on the seafloor as additional reef habitat, (c. Toppling) explosives are detonated to sever the conductors in the middle and pilings and support legs on three sides of the platform at the seafloor and the whole structure is bent over to remain in a horizontal orientation on the seafloor.

Greater than 7,500 oil and gas platforms around the world [1] will need to be decommissioned in the coming decades [2]. Decommissioning is the process by which the fate of these structures is determined once they become uneconomical to operate. This process may encompass one of four alternatives: complete removal, tow-and-place, partial removal (i.e., “topping”), or toppling (laying the structure on its side) (Fig 1) [2–4]. At least 188 decommissioned platforms in the Gulf of Mexico have remained in the ocean to continue functioning as man-made reef habitat since 1947. The ecological impact assessment of these structures (e.g., [5, 6]) has been somewhat limited relative to the research performed on the biological communities associated with platforms off the California coast. This is likely due to less controversy associated with the process in the Gulf of Mexico region, resulting in less societal need for the associated scientific information [3].

With the passage of AB 2503 The California Marine Resources Legacy Act in 2010, the State of California will allow consideration of the partial removal of decommissioned offshore oil platforms as an alternative to complete removal if specified criteria are met. One of these criteria is a finding that conversion to an man-made reef would provide a “net benefit” to the environment as compared to removal of the facility [7]. The determination of what constitutes a “net benefit” is still under consideration, and therefore there is a critical need to understand the biological productivity of these structures and how partial removal may impact associated processes [2–4, 8–14]. Fowler et al. [4] evaluated one of the platforms off the coast of California (Platform Grace) as a case-study of their proposed “multi-criteria decision approach” to determine a preferred decommissioning option. During this process ‘production of exploitable biomass’ and ‘provision of reef habitat’ were ranked by expert opinion as the most important criteria in the decision for this platform. Therefore, 1) given the quantity of biological information now available for platforms in California (e.g., [3, 9, 15–17]) and 2) the likelihood that the Pacific may be the first region where platforms in deeper water are going to be decommissioned [3], the process in California has an opportunity to serve as a model for decommissioning elsewhere.

Secondary (i.e., heterotrophic or animal) production is the sum of new biomass from growth for all individuals in a given area during a unit of time [18, 19]. It is a main pathway of energy flow through an ecosystem as it makes energy available to consumers, including humans [20, 21]. Some of the original motivations for understanding biological productivity stem from the need to estimate the annual biomass of fishes that can be taken from a body of water [18]. Applying a model of annual fish production based on fisheries-independent density and size structure data of fishes from visual surveys, Claisse et al. [22] found that oil and gas platforms off the coast of California have the highest secondary fish production per unit area of seafloor of any marine ecosystem for which similar estimates exist. These high rates of fish production ultimately result from high levels of larval and pelagic juvenile settlement and subsequent growth of primarily rockfishes (genus *Sebastes)* to the substantial amount of complex hardscape habitat created by the platform structure distributed throughout the water column.

Of the two decommissioning options predominantly being considered in California, only partial removal (the other being complete removal) would allow the remaining structure to continue functioning as a reef. In the U.S., partial removal of platforms has typically removed the platform structure down to a depth of 85 ft (26 m) in order to maximize safe navigation, allow for non-use of buoys to mark the location, and reduce unnecessary aids to navigation [23]. Often referred to as “Rigs-to-Reefs,” this terminology is a misnomer since the complex hardscape habitat created by the platform structure distributed throughout the water column already functions as very productive habitat for invertebrates [24–26] and fishes [9, 17, 22] while energy extraction is occurring. However, how partial removal may impact these ecological processes is still undetermined.

Shell mounds are biogenic reefs created by an accumulation of shells (mostly mussels: *Mytilus californianus* and *M*. *galloprovincialis*) that have fallen from the shallow areas of these platforms in California [15, 27]. In addition to creating hard substrate on the otherwise soft-bottom seafloor, the ‘‘faunal litterfall” from the upper portion of the platforms also provides food resources to the benthos under the platforms [27]. The fish communities on a shell mound are typically more similar to the community on the base of the adjacent platform than to those on other shell mounds surrounding different platforms. However, fishes on shell mounds tend to be smaller and less dense than those on platform bases [15]. Subsequent to partial removal we would expect a reduction in the habitat complexity associated with shell mounds on the seafloor surrounding the base of platforms. A thick layer of dozens of sessile invertebrate taxa, including barnacles, sponges, anemones and mussels, covers the submerged platform structure [28]. Mussels are the dominant species from the surface down to around 15 m depth on the platforms, although they occur less frequently down to around 40 m ([29]; observations of the authors). Given this depth range, the mussel’s habitat would be almost non-existent on platforms after partial removal down to 26 m depth. This would result in a decrease in the food subsidy the falling mussels provide [27]. Further, the thickness and the complexity of the shell mounds would also be reduced over time without a continued input of new shells assuming that a platform resided in a depositional area where sedimentation rate surpassed flushing rate. Therefore, the impacts of partial removal should also consider the potential loss of the fish biomass and production associated with the shell mound habitat.

**Fig 2:**
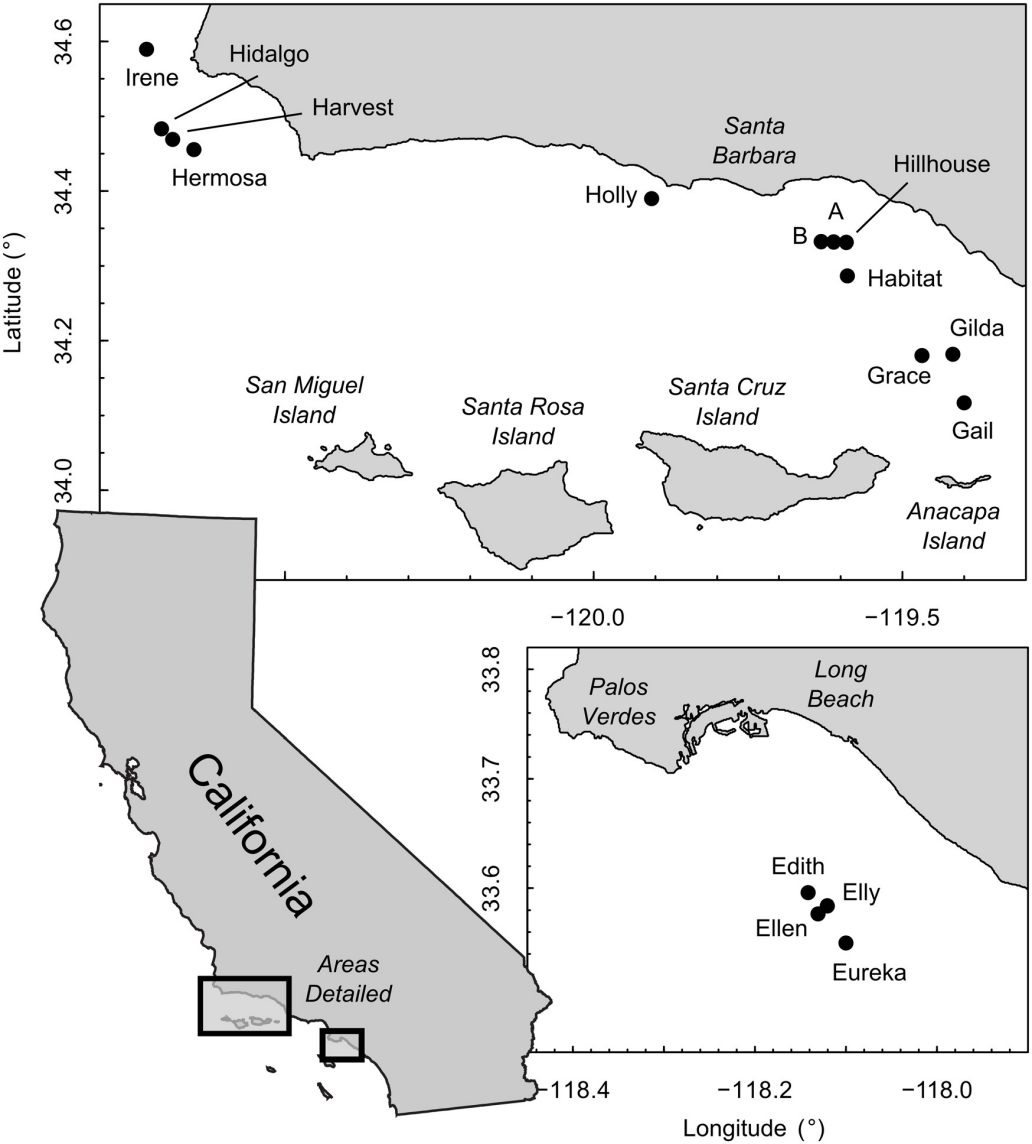
Map of the study area. The 16 oil and gas platforms (filled circles) used in the study were surveyed for at least 5 (up to 15) years between 1995 and 2011.

In the present study we evaluate the potential effects of partial removal on the standing stock biomass (SSB) and annual secondary production of the fish communities living on 16 platforms off the California coast (Fig 2). We calculate 1) the overall SSB (kg) and 2) production (kg/yr) for each complete platform. We then predict the percentage of each that will remain after partial removal by recalculating values with the habitat structure and the associated fishes observed from the water surface to 26 m depth removed from the model. The SSB and fish production is also calculated separately for the shell mound habitats that surround some of these platforms in order to evaluate additional potential impacts associated with a reduction in these habitats after partial removal.

## Materials and Methods

### Data set

Data for this study were obtained from annual visual surveys conducted during daylight hours in the fall between September and November using the manned *Delta* research submersible from 1995 through 2009 and the *Dual Deepworker* from 2010 through 2011. A researcher aboard identified, counted and estimated the total length (TL; to the nearest 5 cm) of all fishes along 2 m wide belt transects. These data are available for download here http://dx.doi.org/10.6084/m9.figshare.1501507. Since different subsets of platforms were surveyed each year, we used data from the 16 platforms (Fig 2) that had been surveyed for at least 5 years, some of which had been surveyed up to 15 years (Table 1). Transects ran along each of the horizontal beams of the platforms from near-surface waters to, in most instances, the bottom. Because horizontal beam length increases with depth, survey effort is roughly proportional to the surface area of the structure at each depth. Transects were classified into three habitat sub-types: “platform shallow habitat”, from the water surface to 26 m depth (i.e., partial removal depth), “platform midwater habitat,” from 26 m depth to 2 m above the seafloor, and “platform base habitat,” the bottom 2 m of the platform [9]. Further details on the survey methodology and platform descriptions are available elsewhere [9, 17]. Annual densities (fish/m^2^) at each platform for each 5 cm size class of each taxon were calculated for each habitat sub-type (i.e., shallow, midwater, base). Transient, highly mobile species (e.g., Jack Mackerel, *Trachurus symmetricus*, Pacific Sardine, *Sardinops sagax*) were excluded from the data set.

**Table 1:** Survey statistics and platform structural dimensions. No.: number of years surveyed. Length: Average total length of transects from annual surveys. Platform statistics: seafloor Depth, estimated surface area of platform structure for each platform habitat sub-type (shallow, midwater, base) and the surface area of seafloor beneath the “footprint” of the platform [47].

Platform		Survey				Platform		
	Level	No.	Length (m)	Min. Depth (m)	Max. Depth (m)	Depth (m)	Surface Area (m^2^)	Footprint Area (m^2^)
**Irene**	shallow						3537	
	midwater	11	193	28	50		10706	
	base	11	207	72	72	74	621	2664
**Hidalgo**	shallow						9402	
	midwater	10	600	32	105		62227	
	base	10	264	129	129	131	1662	4333
**Harvest**	shallow	1	164	20	20		4455	
	midwater	6	966	38	170		73122	
	base	5	316	202	202	204	1544	5890
**Hermosa**	shallow						6018	
	midwater	6	896	41	156		77766	
	base	6	262	179	179	183	1319	5203
**Holly**	shallow	7	85	7	20		6388^a^	
	midwater	13	246	32	35		14043^a^	
	base	11	186	60	60	64	984^a^	1952^a^
**B**	shallow	5	189	5	20		7469	
	midwater	5	312	30	40		13335	
	base					57	1129	1979
**A**	shallow	6	180	5	20		7671	
	midwater	7	266	29	32		13325	
	base					57	1116	1890
**Hillhouse**	shallow	5	214	5	20		7501^a^	
	midwater	5	161	35	35		13705^a^	
	base					58	1141^a^	2014
**Habitat**	shallow	5	192	10	25		4150	
	midwater	5	335	40	65		21616	
	base					92	967	2242
**Gilda**	shallow	5	148	7	25		6035	
	midwater	7	142	39	41		12591	
	base	5	195	56	62	64	862	2081
**Grace**	shallow	2	97	20	25		4789	
	midwater	14	587	25	80		20279	
	base	13	246	92	95	97	777	3004
**Gail**	shallow	2	183	10	10		5156	
	midwater	15	1581	30	168		99596	
	base	14	300	220	224	225	1675	5390
**Edith**	shallow	6	114	10	12		8304	
	midwater	7	169	27	30		8056	
	base	8	212	47	47	49	846	2590
**Elly**	shallow	6	117	12	14		3187^a^	
	midwater	7	297	33	55		10663^a^	
	base	7	220	75	75	78	568^a^	2664^a^
**Ellen**	shallow	5	92	12	14		5930^a^	
	midwater	7	265	30	55		20849^a^	
	base	7	203	77	77	81	1064^a^	2664^a^
**Eureka**	shallow	4	153	15	16		5615^a^	
	midwater	7	1446	35	190		101459^a^	
	base	3	281	210	215	213	1809^a^	5390^a^

^a^When platform dimensions or surface area estimates were unavailable [47], the following proxies were used from platforms with similar structures from similar water depths: Irene for Ellen and Elly surface and base platform dimensions, Gail for Eureka surface and base platform dimensions, C for Holly surface area and surface and base platform dimensions, and A for Hillhouse surface area and surface platform dimensions.

Oil and gas platforms are immense industrial facilities that function continuously throughout the year and the annual surveys were completed at all platforms while they were fully operational. Access and permission to conduct the surveys required a lengthy process with each platform operator (six companies operate the 23 oil and gas production platforms off the southern California coast: ExxonMobil Corporation; Plains Exploration & Production Company; Pacific Operators Offshore, LLC; Beta Operating Company, LLC; Venoco, Inc.; and DCOR, LLC). This process involved a detailed Research Execution and Emergency Communication Plan for each annual cruise, individual legal liability waivers for each platform signed by all personnel on the ship, and additional information as requested by each separate operator. Upon arrival at the platform, the platform superintendent from the operators listed above would issue the final clearance to dive on the day of the survey. Institutional Animal Care and Use Committee (IACUC) approval was not required from the relevant institutions for this study based on the methods used. Fishes, including endangered or protected species, were not collected, nor otherwise handled for this study.

### Platform Biological Metrics

**Fig 3:**
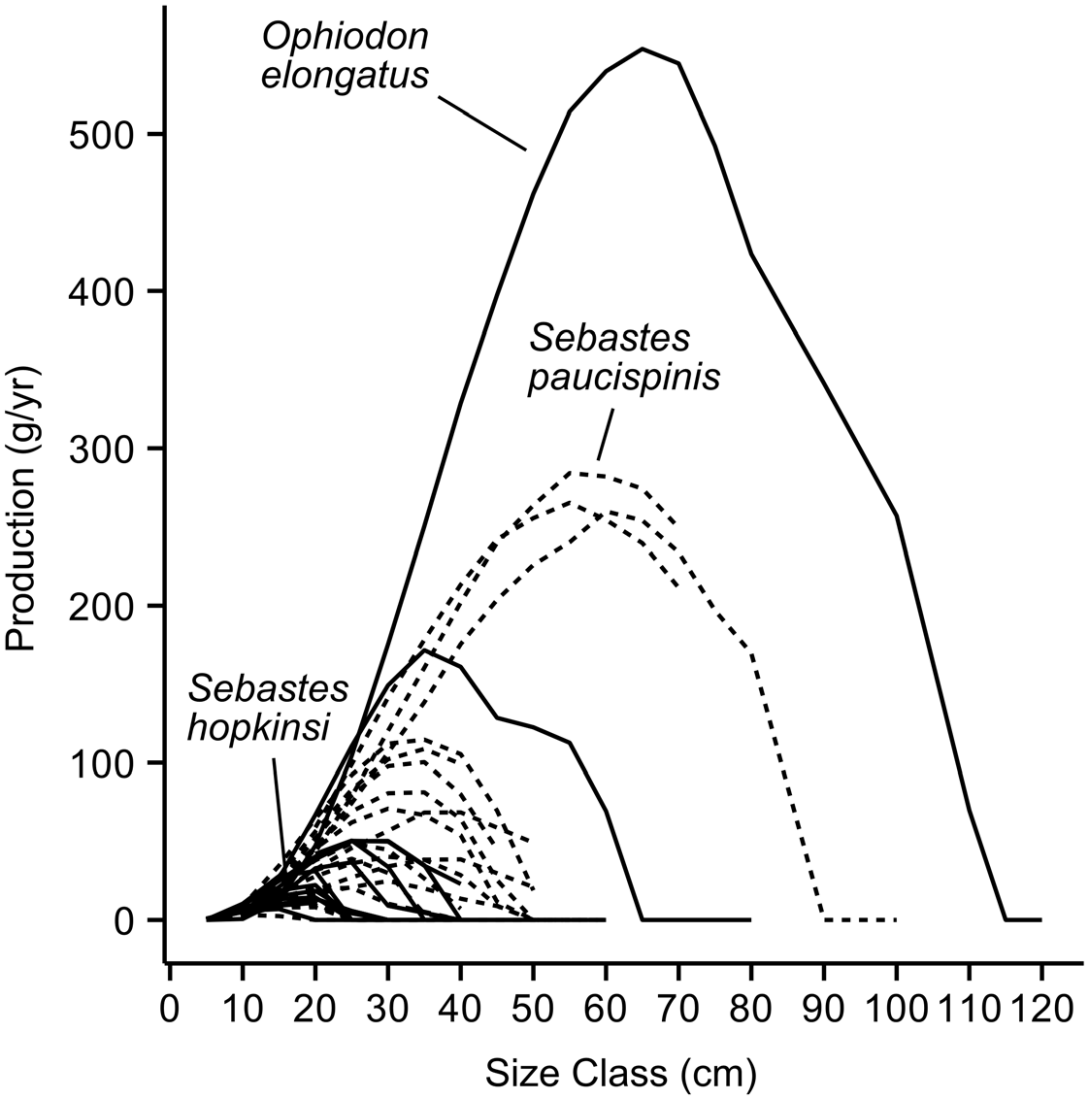
Annual somatic production per individual observed by size class. The values presented here are the product of the annual growth in weight and annual survivorship (see reference [22] for more detail) and plotted for each species that contributed at least 1% of Total Production on any platform (S2 Table). Values were plotted over the size classes that a species was observed and rockfishes, *Sebastes spp*. were plotted with dashed lines. We also identify the curves for the two species observed that have the highest individual production rates, and for *Sebastes hopkinsi* which was used as the proxy for unidentified rockfishes because it was the most common species and its relatively low annual production rate per individual would result in a relatively conservative production estimate. Note that while growth in length according to the von Bertalanffy growth equation is highest at the smallest size, production here is maximized at intermediate lengths due to the exponential increase with weight-at-length and low survival rates at small sizes. Also, production goes to 0 when fishes grow larger than the mean asymptotic length predicted by the von Bertalanffy growth function [22, 30].

We first calculated total standing stock biomass (SSB) of the fish community for each platform habitat sub-type (i.e., shallow, midwater, base) during each year surveyed. Observed fish lengths were converted to biomass using species-specific weight-at-length and (when necessary) length-length conversion relationships from the literature (see Table S3 in reference [22]). We also calculate the annual secondary production for the fish community (i.e., “Total Production”) based on a previously developed model (for a detailed description of the model see reference [22]). The model defines Total Production of the fish community as the sum of two components. The first being “Somatic Production,” which is the difference between the observed biomass during surveys and the biomass predicted one year later. The size- and species-specific one-year increase in fish length is predicted using the Fabens version of the von Bertalanffy growth function [30], a standard method for modeling fish growth rates. Biomass is calculated using the previously mentioned species-specific morphometric relationships. The production from fishes that do not survive the one-year time interval are not included in the production estimate. Annual survivorship rates are incorporated in the model using the size- and species-specific mortality function described in Gislason et al. [31]. The second component of Total Production is “Recruitment Production” which estimates production from the growth of post larval and pelagic juvenile fishes that settled or immigrated during the one year time interval and survived up to the time of survey (following reference [32]). In some cases fishes were not identified to species during surveys (see Table S3 in reference [22]). For the most common of these cases, unidentified rockfishes (*Sebastes spp*.), we chose to use Squarespot Rockfish, *Sebastes hopkinsi*, as a proxy because it was the most frequently observed species across all surveys and, as a relatively small-bodied rockfish with a relatively low annual production per individual (Fig 3), it would result in a more conservative production estimate.

To evaluate the impacts of partial removal, annual metrics were calculated for each “complete platform” and “partially removed” platform. This was done by multiplying the SSB and Total Production density metrics (per m^2^ of platform structure) by the submerged surface area of platform structure for each platform habitat sub-type (i.e., shallow, midwater, base) (Table 2). Complete platform metrics included all three platform habitat sub-types, while partially removed platform metrics only included the midwater and base habitats. The amount of platform structural surface area in each habitat sub-type was allocated in proportion to the volume in each habitat type, calculated from platform dimensions using the formula for a truncated-pyramid [33]. If the platform base or shallow habitat could not be sampled during a given year (Table 1), typically due to limited visibility or sea conditions near the surface, the mean of its available annual values were used. Since platform base habitat was never surveyed for Platforms A, B, Hillhouse and Habitat, the mean platform base values from Holly were used as a proxy given its geographic proximity and habitat similarity. This was chosen as a better alternative than applying the midwater density values from the respective platforms to their base habitats because of the substantial differences in species composition and size structure of the fish assemblages between base and midwater habitats [9, 22]. Since platform shallow habitat was never surveyed (or only once) for the northernmost platforms (Irene, Hidalgo, Harvest and Hermosa; Table 1), the platform shallow values from Holly, B, A, Hillhouse, and Habitat were averaged and used as a proxy given their geographic proximity. It is likely however that these shallow habitat proxy values are going to be higher than the low densities of fishes which have been observed on these northernmost platforms at shallow depths ([16]; observations of the authors). Therefore, this approach is thought to be conservative with respect to not underestimating the impact of partial removal on these northernmost platforms.

**Table 2:** Surface area and mean of annual values for platform habitat sub-types and shell mounds.

Platform	Habitat Sub-types	Surface Area (m^2^)	SSB Density (kg/m^2^)	SSB (kg)	Total Production Density (kg/m^2^/yr)	Total Production (kg/yr)
Irene	shallow	3537	0.118	419	0.091	323
	midwater	10706	0.118	1267	0.091	978
	base	621	0.305	189	0.068	42
	shell mound	13484	0.041	555	0.024	324
Hidalgo	shallow	9402	0.015	138	0.017	163
	midwater	62227	0.015	911	0.017	1080
	base	1662	0.221	367	0.032	54
	shell mound		0.022		0.008	
Harvest	shallow	4455	0.018	78	0.012	54
	midwater	73122	0.021	1520	0.013	962
	base	1544	0.072	112	0.010	16
	shell mound		0.019		0.004	
Hermosa	shallow	6018	0.033	200	0.020	118
	midwater	77766	0.033	2584	0.020	1531
	base	1319	0.126	166	0.013	17
	shell mound	642	0.048	31	0.007	4
Holly	shallow	6388	0.031	197	0.008	53
	midwater	14043	0.038	536	0.017	241
	base	984	0.283	279	0.040	39
	shell mound		0.053		0.009	
B	shallow	7469	0.022	162	0.002	15
	midwater	13335	0.036	475	0.016	209
	base	1129	0.158	178	0.028	31
A	shallow	7671	0.046	355	0.005	38
	midwater	13325	0.047	625	0.013	172
	base	1116	0.158	176	0.028	31
Hillhouse	shallow	7501	0.022	164	0.005	40
	midwater	13705	0.049	675	0.047	649
	base	1141	0.158	180	0.028	32
Habitat	shallow	4150	0.006	26	0.002	8
	midwater	21616	0.057	1222	0.023	495
	base	967	0.158	153	0.028	27
Gilda	shallow	6035	0.014	86	0.005	27
	midwater	12591	0.027	337	0.021	268
	base	862	0.345	297	0.162	140
	shell mound	18290	0.139	2534	0.068	1253
Grace	shallow	4789	0.132	631	0.058	276
	midwater	20279	0.163	3296	0.077	1563
	base	777	0.424	329	0.055	43
	shell mound	22754	0.073	1655	0.010	238
Gail	shallow	5156	0.013	69	0.005	25
	midwater	99596	0.004	419	0.004	414
	base	1675	0.447	749	0.070	117
	shell mound	655	0.031	20	0.005	3
Edith	shallow	8304	0.189	1570	0.038	315
	midwater	8056	0.028	227	0.004	34
	base	846	0.150	127	0.075	64
	shell mound		0.114		0.030	
Elly	shallow	3187	0.100	318	0.015	46
	midwater	10663	0.240	2563	0.066	704
	base	568	0.688	391	0.108	61
	shell mound		0.176		0.037	
Ellen	shallow	5930	0.070	414	0.019	115
	midwater	20849	0.283	5892	0.108	2243
	base	1064	0.408	434	0.057	61
	shell mound		0.100		0.016	
Eureka	shallow	5615	0.103	578	0.030	166
	midwater	101459	0.105	10679	0.035	3540
	base	1809	0.116	211	0.010	18
	shell mound		0.005		0.001	

Standing Stock Biomass (SSB) density (kg/m^2^) and Total Production density (kg/m^2^/yr) metrics (scaled per m^2^ of platform structure or shell mound) are multiplied by the habitat surface area to yield the overall SSB (kg) or Total Production (kg/yr) estimates for the platform structure in each depth range or habitat. Shell mound surface area estimates were only available for some platforms [34], and their areal extent around the remaining platforms is currently unknown.

All biological metrics were also reported as densities per m^2^ of seafloor. These were calculated by dividing the overall values for a complete for partially removed platform by the surface area of seafloor beneath the footprint of the platform. This was done so that these results could be directly compared with previous fish production estimates in the literature scaled in this manner. These include estimates of secondary fish production from these oil platforms [22] and from other marine ecosystems (e.g., those in Table 1 in reference [22]).

### Shell Mound Biological Metrics

Partial removal will likely result in a reduction over time in the thickness and complexity of shell mound habitats surrounding platforms and in the food subsidy associated with falling invertebrates [27], including a possible complete loss of this habitat. Therefore, the previously described biological metrics were also calculated for this habitat type to estimate the maximum potential associated losses. Shell mound habitats were typically surveyed during annual platform surveys as previously described (Table 3). Separate 2 m wide belt transects were performed across the shell mound habitats surrounding some platforms. These transects did not overlap with those surveying the platform base habitats (for further description of shell mound habitats see reference [15]). The surface area of the shell mounds associated with a platform [34] was also available in some cases (Table 3). Where available, the surface area was multiplied by the annual per m^2^ scaled metrics (Table 2) to yield overall estimates of SSB and Total Production for the entire shell mound habitat surrounding a given platform.

**Table 3:** Shell mound survey statistics and area. No.: number of years surveyed. Length: Average total length of transects from annual surveys. Minimum and maximum depths of the transects across the shell mounds. Note that only some of the shell mounds associated with the platforms were surveyed for fishes, and some areal extent estimates of the shell mounds [34] were not available (na).

Platform	No.	Length (m)	Min. Depth (m)	Max. Depth (m)	Shell mound Area (m^2^)
**Irene**	10	246	72	72	13484
**Hidalgo**	9	320	128	129	na
**Harvest**	5	493	202	203	na
**Hermosa**	5	251	179	179	642
**Holly**	6	188	59	62	na
**Gilda**	5	238	56	62	18290
**Grace**	14	300	92	92	22754
**Gail**	13	366	220	224	655
**Edith**	8	210	47	47	na
**Elly**	7	265	75	75	na
**Ellen**	7	276	77	77	na
**Eureka**	3	390	210	216	na

## Results

### Platform SSB and Total Production

**Fig 4:**
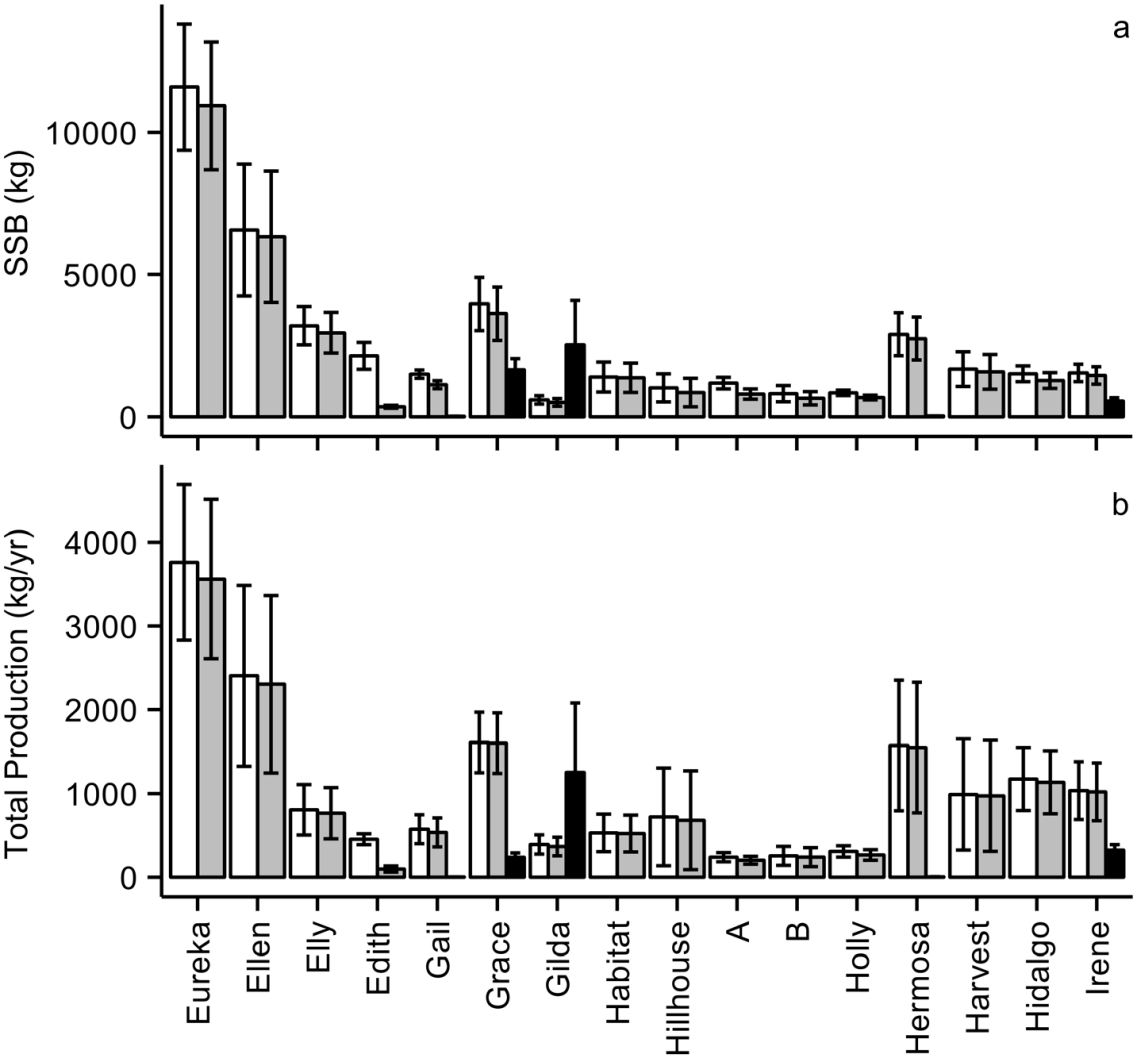
(a) Standing Stock Biomass (SSB) and (b) Total Production with SE error bars for complete platforms (white bars), partially removed platforms (gray bars), and for the entire shell mounds associated with some platforms (black bars). Gray bars represent the predicted overall biomass or Total Production that will be retained on the remaining platform structure after partial removal. While the fate of shell mound habitats after partial removal is currently unknown, the black bars represent a potential additional reduction in SSB and production if they were totally lost. Note that both a shell mound surface area estimate and associated fish survey data (permitting overall SSB and Total Production estimates) were only available for the five platforms with black bars shown. It should not be assumed that shell mounds are not present around some platforms because estimates are not provided here. Platforms are ordered from south to north (Fig 2).

Mean annual SSB and Total Production for complete platforms was highly variable, spanning an order of magnitude across platforms (Fig 4; S1 Table). SSB ranged from 11,585 kg on Platform Eureka to 816 kg on Platform B. Total Production ranged from 3759 kg/yr on Platform Eureka to 240 kg/yr on Platform A. Relatively few taxa, largely rockfishes and Lingcod, *Ophiodon elongatus*, contributed the majority of SSB and Total Production on any given platform. While the top contributors for individual platforms varied, typically only one to three species accounted for more than two-thirds of the Total Production on any platform (S2 Table).

**Fig 5:**
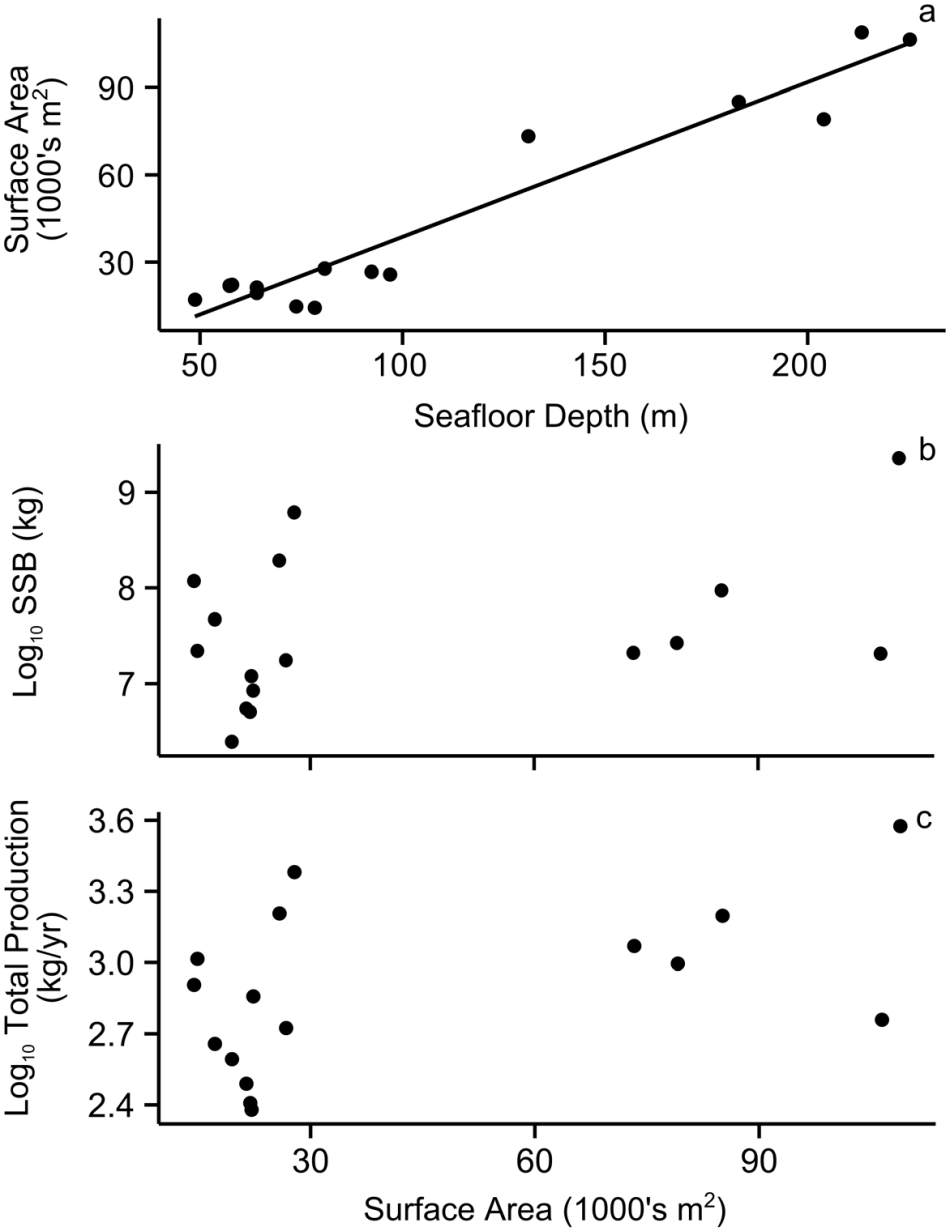
(a) The relationship between seafloor depth and platform submerged surface area, and the relationships between platform submerged surface area and (b) Log_10_ complete platform standing stock biomass (SSB) or (c) Log_10_ complete platform Total Production. Depth was significantly related to platform surface area [Surface Area (m^2^) = 531 * Seafloor Depth (m)-14464; R^2^ = 0.93; p-value < 0.001]. There was no significant linear relationship between platform surface area and complete platform Log_10_ SSB (R^2^ = 0.09; p-value = 0.141), nor between platform surface area and complete platform Log_10_ Total Production (R^2^ = 0.19; p-value = 0.053).

The surface area of the platform structure available as fish habitat is dependent on seafloor depth, but was not a good predictor of complete platform SSB or Total Production. As expected, there was a clear relationship with the seafloor depth and submerged surface area of the platform structure [Fig 5a; Surface Area (m^2^) = 531 * Seafloor Depth (m)-14464; R^2^ = 0.93; F = 185.2; DF_1,14_; p-value < 0.001]. However, there was no significant linear relationship between platform surface area and complete platform Log_10_ SSB (Fig 5b; R^2^ = 0.09; p-value = 0.141), nor between platform surface area and complete platform Log_10_ Total Production (Fig 5c; R^2^ = 0.19; p-value = 0.053).

The impact of partial removal would be limited on all but one of the platforms examined. On average, 80% of SSB and 86% of Total Production would be retained after partial removal, with above 90% retention expected for many platforms for both metrics (Fig 4, S1 and S3 Tables). Platform Edith, located in the southern end of the geographical range of platforms in our study (Fig 2), was the lone exception retaining only 18.7% of SSB and 20.1% of Total Production (Fig 4, S1 Table). It was also an exception in that Blacksmith, *Chromis punctipinnis*, a primarily planktivorous damselfish was the top contributor for Platform Edith, providing 53.8% of SSB and 63.9% of Total Production (S2 Table).

### Shell mound SSB and Total Production

The shell mounds associated with 12 of the platforms were surveyed permitting calculation of density scaled biological metrics for those habitats (Table 3). The SSB density and Total Production density on shell mounds varied considerably across sites (Table 2, S4 Table). SSB density ranged from 139 g/m^2^ on shell mounds associated with Platform Gilda to 4.93 g/m^2^ on shell mounds associated with Platform Eureka. Total Production density ranged from 68 g/m^2^/yr on shell mounds associated with Platform Gilda to 0.8 g/m^2^/yr on shell mounds associated with Platform Eureka (S4 Table). Lingcod was one of the top two contributors to Total Production at the shell mounds surrounding all but two of the twelve platforms where shell mounds were surveyed (S5 Table).

Estimates of the areal extent of the shell mounds were available for five platforms for which we also had shell mound fish survey data (Tables 2 and 3) permitting estimation of the overall SSB and Total Production for the entire shell mound. The three shell mounds with relatively large areal extents (Irene, Gilda, Grace; Table 3), comparable to the total surface area of some complete platforms (Table 1), had overall SSB and Total Production estimates (Fig 4, Table 2, S6 Table) that were similar to some platforms (Fig 4, S1 Table). The other two shell mounds covered small areas of seafloor (Hermosa 642 m^2^, Gail 655 m^2^) and had very low estimates of overall SSB and Total Production (Fig 4, Table 2, S6 Table).

## Discussion

While the SSB and Total Production of fishes of complete platforms varied substantially across platforms, a high percentage of both will likely be retained after partial removal on almost all platforms off of the coast of California. Further, partially removed platforms would still have some of the highest production values (when scaled to per m^2^ of seafloor) of any marine habitat globally (platform Total Production after partial removal range: 37.8 to 865.1 g/m^2^/yr, S3 Table; fish production in other habitats range: 0.9 to 74.2 g/m^2^/yr, see Table 1 in reference [22]). Many of the rockfishes that make up a substantial proportion of the biomass and production on platforms are important to recreational and commercial fisheries, and two, Bocaccio, *Sebastes paucispinis*, and Widow Rockfish, *S*. *entomelas*, are currently managed under federal rebuilding plans [35]. These results suggest that partially removed platforms will still remain viable habitats for these important species.

Recruitment of most species of larval and pelagic juvenile rockfishes to platform habitat, the ultimate driver of both the somatic and recruitment components of Total Production [22], appears unlikely to be impacted substantially by partial removal. Love et al. [16] concluded that recruitment of rockfishes does not appear dependent upon the platform structure extending up to the surface. They found that young-of-the-year (YOY) fish assemblages on the platform structure at depths that would remain after partial removal (26–35 m; classified here as midwater), were similar to those observed on deeper pinnacle reefs and shipwrecks (structures not reaching the surface). These assemblages were dominated by the rockfishes we found to be the major contributors to Total Production on almost all platforms in the present study (i.e., Bocaccio, Shortbelly Rockfish, *S*. *jordani*, Widow Rockfish, and Squarespot Rockfish; S2 Table). Carr et al. [36] also found that YOY of these species were observed primarily at the midwater depths with relatively few above 26 m. While they did find YOY of a few rockfish species (i.e., Copper Rockfish, *S*. *caurinus*, Kelp Rockfish, *S*. *atrovirens*, Gopher Rockfish, *S*. *carnatus and* Black-and-Yellow Rockfish, *S*. *chrysomelas*) were residing in the highest densities on platform structure around 8 m depth, these species were not major contributors to our production estimates for complete platforms. Further, these shallow water rockfishes typically recruit to and reside in nearshore kelp forest and rocky reef habitats which are abundant along the near-shore California coast [37, 38]. Therefore, the loss of the shallow platform habitat from partial removal would likely have a minor impact on their populations. Of these species, the maximum contribution was made by Copper Rockfish on Platform Holly, contributing 6.9% of Total Production. Typically they contributed less than 1% of the overall fish production or SSB on the platforms we examined. Generally we would not expect to see substantial reductions in overall rates of secondary production nor SBB of rockfishes as a result of changes in recruitment after partial removal.

We expect that the primary impact from partial removal would be a reduction of SSB and production of the typically shallow-dwelling nearshore species that reside as adults in shallow platform habitats. The loss of these shallow dwelling species was reflected in Martin and Lowe [8], where SCUBA surveys were used to evaluate the fish community structure down to 30 m depth on the group of platforms located at the southern end of our study area (platforms in federal waters starting with the letter E in Fig 2, plus Platforms Esther and Eva in state waters). They report that partial removal would potentially result in the retention of only 5% of the total fish density and 23% of the total fish biomass. At these shallow depths, the common species they observed included California Sheephead, *Semicossyphus pulcher*, Blacksmith, Garibaldi, *Hypsypops rubicundus*, Opaleye *Girella nigricans*, and Kelp Bass *Paralabrax clathratus*. However, while our results were similar for Platform Edith (only 25% of SSB and Total Production retained), our results for Platforms Elly, Ellen and Eureka show a much more limited impact of partial removal with 84.8%, 91.7% and 94.2% of Total Production retained, respectively. At these sites, we were able to account for fishes living below SCUBA depth on the platform structure down to the seafloor, and the majority of the SSB and Total Production on these platforms were due to deeper-dwelling rockfishes (S2 Table). On Platform Edith, Blacksmith were observed almost entirely above 26 m and contributed the majority of the SSB and Total Production (53.8% and 63.9%, respectively). Therefore, while the loss of fishes typically found residing in the upper portions of the platform structure should be considered in an evaluation of platform decommissioning options [8, 36], they likely only represent a small proportion of the fishes living associated with most of these platforms off of California.

The shell mounds, which surround some platforms in our study, were moderately productive fish habitats, although the areal extent of these structures varied greatly. Shell mound Total Production density values (0.8 to 68 g/m^2^/yr; Table 2, S6 Table) were similar, or in some cases were much greater than, those from deep natural rocky reefs in the region located at similar depths (4.4 to 22.4 g/m^2^/yr, see Table 1 in reference [22]). The overall annual amount of Total Production for entire shell mounds can be quite substantial when they cover large areas (Table 3), equivalent to that of a low to moderately productive complete platform (Fig 4b). Therefore, if partial removal results in a decline in the complexity and areal extent of these larger shell mound habitats over time, it could also cause a loss in the shell mound associated fish SSB and production. Further, it is not clear how much the food subsidies provided by the faunal litterfall to these habitats [27] would impact the associated production of fishes even if the physical structure of the shell mounds remained.

While the ultimate fate of shell mound habitats after partial removal is currently unknown, we can consider what additional reductions in fish production would occur if they were lost completely. In the case of platform Gilda, with one of the largest shell mounds (over 18,000 m^2^), it would mean a 76% reduction in the combined overall Total Production of the platform and shell mound (Fig 4b). However, this is an extreme example since Gilda had the most productive shell mound of any we examined, about double that of the remaining highly productive shell mounds (S4 Table). The two other platforms with large shell mounds illustrate more moderate potential reductions if the shell mounds are lost. Platform Grace, which has the largest shell mound area of those included in this study (almost 23,000 m^2^), would only have a 13% decline in production. While the loss of the shell mound associated with platform Irene (around 13,000 m^2^) would yield a 24% reduction. For platforms with very small shell mounds (e.g., Hermosa and Gail at around 650 m^2^), the loss in Total Production would be negligible (0.3% and 0.5%, respectively). Finally, it is important to note that reductions of these productive habitats under a complete platform removal decommissioning option (e.g., [27]) would be similar to, or even greater than, those under a partial removal scenario.

Options do exist to enhance or augment the habitat on the seafloor around the base of partially removed platforms. Larger and older rockfishes of many species tend to move deeper as they grow [36, 39, 40]. Those on platforms are able to take refuge in complex sheltering habitats created by the large horizontal beams typically at or near the seafloor at the base of a platform [41]. Given that in California the platform base habitat (bottom 2 m) has the highest production rate of any platform sub-habitat type per unit area [22], adding additional structure at the seafloor will likely have positive impacts on production. Seafloor habitats can be augmented by placing the partially removed platform superstructure or some other additional habitat enrichment material (e.g., quarry rock or pieces of concrete) adjacent to the platform base [2, 3, 9]. Rock boulders have been placed around the bases of monopile offshore wind turbines to prevent erosion or scour of soft sediments, and they subsequently were found to create nursery habitat for commercially important fishery species [14, 42]. Some or all of the superstructure of decommissioned platforms has been placed on the seafloor adjacent to the platform base in the Gulf of Mexico and the east coast of Florida [3, 43]. A critical consideration when doing this is the final orientation of crossbeams or other structures relative to the seafloor, as this greatly influences the performance of these habitats [41]. Habitat augmentation after partial removal would maximize the potential for YOY fishes to eventually populate the new structure as they matured, taking advantage of the positive effects of the nursery recruitment habitat located through the midwater portion of the remaining platform structure [9, 22]. This may have the potential to mitigate reductions in production associated with removing platform structure in the surface waters and the potential reduction in the extent of shell mound habitats around some platforms.

Overall SSB or Total Production was highly variable across the platforms off the California coast (Fig 4), but neither seafloor depth nor total submerged platform surface area appears to be a sufficient proxy for estimating these metrics (Fig 5). As an example, Platform Eureka had the highest SSB and Total Production by far. This can be partially attributable to its large submerged surface area, the 2^nd^ highest of the platforms in our study (103,268 m^2^; Table 1). However, Platform Gail, with the largest submerged surface area in our study (106,427 m^2^), was on the lower end in terms of SSB and Total Production (Fig 4). This unexplained variation thus creates an opportunity in future studies to examine if differences in structural design and/or geographic location make one platform more productive than another [6, 22].

Decisions related to the appropriate decommissioning option for individual platforms in California should consider the magnitude of the net benefit to the environment that the remaining platform structure would provide as compared to complete removal [7]. The platform decommissioning process is complicated and should take into account multiple criteria related to the interests of many stakeholders [4, 44]. Our estimates of the SSB and Total Production retained after partial removal can contribute to this process by being considered one element of net benefit provided by choosing partial over complete removal, with even greater benefits expected if the seafloor habitat surrounding the base of platforms is augmented with additional structure. Complete platform removal is typically done by detonating explosives 5 m below the seafloor to sever the well conductors, platform anchor pilings, and support legs. The use of explosives results in the mortality of most fishes associated with the platform [45], effectively eliminating its entire SSB. Removing the platform structure means any subsequent productive value of platform habitat is also lost, and potentially the production associated with any surrounding shell mounds. While platforms represent a small contribution to the overall subtidal hard substratum in California [13], these structures may be providing a considerable amount of the hard substrate below a depth of 50 meters in the soft-bottom outer shelf regions where they occur [10, 11]. If partial removal is chosen as the preferred decommissioning option, it will be possible to better empirically assess the function of the platform structure (above 26 m depth) extending to the surface related to fish recruitment and biological productivity associated with the platform structure and shell mound habitat that remains [4, 36]. It will be critical that these partially removed platforms are regularly surveyed, likely over a 5 to 10 year period given the high temporal and spatial variability in fish recruitment and subsequent production [22, 46], so that these processes can be more thoroughly understood and applied to future decommissioning decisions.

## Supporting Information

S1 Table Mean of annual values for complete platforms (C) and partially removed (PR) platforms and the percent retained after partial removal (%). Standard errors are in parentheses.
(DOCX)

S2 Table Percent contribution of individual taxa to complete platform metrics. Only taxa that contribute at least 1.0% of the Total Production are included. Taxa are sorted by percent contribution to Total Production. Percentages based on species actually observed on specific platforms, i.e., proxy values used for complete platform metric calculations (in the event a platform sub-habitat could not be sampled during a given year) are not included in these calculations.
(DOCX)

S3 Table Mean of annual values scaled to per m2 of seafloor beneath the platform for complete platforms (C) and partially removed (PR) platforms and the percent retained after partial removal (%). These values are calculated by dividing the overall values for an entire platform (S1 Table) by the surface area of seafloor beneath the footprint of the platform (Table 1). Standard errors are in parentheses.
(DOCX)

S4 Table Shell mound mean (SE) of annual density values per m^2^ of seafloor. (DOCX)

S5 Table Shell mound percent contribution of individual taxa. Only taxa that contribute at least 1.0% of the Total Production are included. Taxa are sorted by percent contribution to Total Production.
(DOCX)

S6 Table Shell mound mean (SE) of annual overall values, i.e., density values multiplied by the total area of the shell mound for a given platform (Table 2). (DOCX)
