# Supplementary material for: Impacts from Partial Removal of Decommissioned Oil and Gas Platforms on Fish Biomass and Production on the Remaining Platform Structure and Surrounding Shell Mounds
Source: PLoS One. 2015 Sep 2;10(9):e0135812. doi: 10.1371/journal.pone.0135812 (PMC4557934; doi:10.1371/journal.pone.0135812)
Supplement: S1 Table — Standard errors are in parentheses. (DOCX) [file pone.0135812.s001.docx]

|  | **SSB (kg)** | | | **Somatic Production (kg/yr)** | | | **Recruitment Production (kg/yr)** | | | **Total Production (kg/yr)** | | |
| --- | --- | --- | --- | --- | --- | --- | --- | --- | --- | --- | --- | --- |
| **Platform** | **C** | **PR** | **%** | **C** | **PR** | **%** | **C** | **PR** | **%** | **C** | **PR** | **%** |
| **Irene** | 1546 | 1456 | 89.9 | 570 | 558 | 93.9 | 466 | 462 | 94.8 | 1035 | 1020 | 95 |
|  | (307) | (307) | (2.6) | (146) | (146) | (2.5) | (240) | (240) | (1.8) | (345) | (345) | (2.2) |
| **Hidalgo** | 1515 | 1278 | 80.2 | 394 | 365 | 87.7 | 779 | 769 | 96.1 | 1173 | 1134 | 92.3 |
|  | (277) | (277) | (3) | (94) | (94) | (3) | (286) | (286) | (1.4) | (376) | (376) | (2.3) |
| **Harvest** | 1680 | 1583 | 90.1 | 459 | 447 | 95.1 | 530 | 526 | 94.2 | 988 | 972 | 95.1 |
|  | (610) | (611) | (3.1) | (195) | (196) | (1.4) | (473) | (473) | (1.8) | (664) | (663) | (1.5) |
| **Hermosa** | 2901 | 2750 | 91.8 | 785 | 766 | 95.5 | 788 | 782 | 94.3 | 1573 | 1548 | 95.7 |
|  | (749) | (749) | (2.9) | (232) | (232) | (1.9) | (587) | (587) | (2.5) | (779) | (779) | (1.8) |
| **Holly** | 845 | 680 | 80.2 | 204 | 169 | 81.7 | 104 | 97 | 83.7 | 308 | 266 | 83.5 |
|  | (95) | (83) | (2.4) | (40) | (35) | (2.7) | (40) | (40) | (4.7) | (68) | (63) | (2.9) |
| **B** | 816 | 654 | 83.3 | 185 | 170 | 90.1 | 70 | 70 | 99.3 | 256 | 240 | 91.4 |
|  | (285) | (235) | (8.6) | (78) | (76) | (6.9) | (57) | (57) | (0.6) | (114) | (113) | (6.2) |
| **A** | 1187 | 801 | 64.4 | 208 | 171 | 80.9 | 32 | 31 | 96.8 | 240 | 203 | 84.7 |
|  | (204) | (180) | (4.3) | (58) | (51) | (3.1) | (14) | (14) | (2.3) | (55) | (47) | (2.2) |
| **Hillhouse** | 1020 | 855 | 78.0 | 321 | 305 | 83.1 | 400 | 376 | 83.1 | 721 | 681 | 81 |
|  | (494) | (501) | (7.9) | (231) | (234) | (7.9) | (353) | (356) | (12.9) | (584) | (590) | (10.5) |
| **Habitat** | 1401 | 1375 | 97.9 | 443 | 438 | 98.5 | 87 | 84 | 98 | 530 | 522 | 98.5 |
|  | (527) | (516) | (0.6) | (200) | (198) | (0.5) | (37) | (35) | (1.3) | (225) | (220) | (0.4) |
| **Gilda** | 597 | 509 | 82.5 | 199 | 185 | 90.5 | 192 | 182 | 93.7 | 392 | 367 | 92.5 |
|  | (148) | (135) | (3.9) | (54) | (52) | (2.6) | (69) | (67) | (2.9) | (115) | (111) | (2.5) |
| **Grace** | 3965 | 3623 | 82.0 | 1252 | 1245 | 97.2 | 356 | 355 | 94.9 | 1609 | 1600 | 97.5 |
|  | (931) | (932) | (4.4) | (357) | (357) | (1.2) | (124) | (124) | (3) | (361) | (361) | (1.2) |
| **Gail** | 1502 | 1132 | 72.8 | 309 | 273 | 84.5 | 266 | 263 | 95.6 | 574 | 536 | 87.9 |
|  | (146) | (141) | (2.4) | (64) | (64) | (1.8) | (112) | (112) | (1) | (173) | (173) | (1.8) |
| **Edith** | 2147 | 354 | 18.7 | 225 | 59 | 27.5 | 229 | 39 | 23.5 | 454 | 98 | 20.1 |
|  | (474) | (52) | (2.1) | (42) | (12) | (3.3) | (65) | (26) | (12.8) | (65) | (37) | (5.2) |
| **Elly** | 3202 | 2954 | 88.3 | 426 | 408 | 91.1 | 380 | 358 | 70.9 | 806 | 766 | 86 |
|  | (667) | (708) | (4.4) | (124) | (126) | (4.2) | (200) | (200) | (13.9) | (301) | (306) | (6.7) |
| **Ellen** | 6563 | 6326 | 92.8 | 1534 | 1512 | 95 | 871 | 793 | 77.9 | 2405 | 2304 | 89.9 |
|  | (2320) | (2312) | (2.2) | (745) | (742) | (2) | (488) | (494) | (9.7) | (1080) | (1060) | (4.8) |
| **Eureka** | 11585 | 10926 | 93.2 | 1908 | 1791 | 92.6 | 1851 | 1768 | 90.4 | 3759 | 3559 | 92.8 |
|  | (2214) | (2241) | (1.4) | (348) | (363) | (1.6) | (610) | (621) | (3.1) | (928) | (951) | (1.5) |

**S1 Table. Mean of annual values for complete platforms (C) and partially removed platforms (PR) and the percent retained after partial removal (%).**

Standard errors are in parentheses.
