# Supplementary material for: Impacts from Partial Removal of Decommissioned Oil and Gas Platforms on Fish Biomass and Production on the Remaining Platform Structure and Surrounding Shell Mounds
Source: PLoS One. 2015 Sep 2;10(9):e0135812. doi: 10.1371/journal.pone.0135812 (PMC4557934; doi:10.1371/journal.pone.0135812)
Supplement: S2 Table — Only taxa that contribute at least 1.0% of the Total Production are included. Taxa are sorted by percent contribution to Total Production. Percentages based on species actually observed on specific platforms, i.e., proxy values used for complete platform metric calculations (in the event a platform sub-habitat could not be sampled during a given year) are not included in these calculations. (DOCX) [file pone.0135812.s002.docx]

**S2 Table. Percent contribution of individual taxa to complete platform metrics.** Only taxa that contribute at least 1.0% of the Total Production are included. Taxa are sorted by percent contribution to Total Production. Percentages based on species actually observed on specific platforms, i.e., proxy values used for complete platform metric calculations (in the event a platform sub-habitat could not be surveyed during a given year) are not included in these calculations.

| **Platform** | **Taxon** | **Biomass** | **Somatic Production** | **Recruitment Production** | **Total Production** |
| --- | --- | --- | --- | --- | --- |
| **Irene** | *Sebastes jordani* | 14.3 | 19.2 | 44.6 | 30.7 |
|  | *Sebastes entomelas* | 46.9 | 54.0 | 1.9 | 30.5 |
|  | *Sebastes spp.* | 15.8 | 11.0 | 29.2 | 19.3 |
|  | *Sebastes paucispinis* | 5.8 | 7.9 | 18.2 | 12.5 |
|  | *Sebastes caurinus* | 5.7 | 3.1 | 0.6 | 2.0 |
|  | *Ophiodon elongatus* | 1.0 | 1.2 | 1.6 | 1.4 |
|  | *Sebastes flavidus* | 0.7 | 0.6 | 1.9 | 1.2 |
| **Hidalgo** | *Sebastes spp.* | 58.8 | 70.3 | 96.6 | 88.1 |
|  | *Sebastes entomelas* | 9.8 | 13.0 | 0.7 | 4.7 |
|  | *Sebastes hopkinsi* | 1.0 | 1.1 | 1.4 | 1.3 |
|  | *Ophiodon elongatus* | 2.8 | 3.0 | 0.2 | 1.1 |
|  | *Sebastes semicinctus* | 7.4 | 3.4 | 0.0 | 1.1 |
| **Harvest** | *Sebastes spp.* | 27.5 | 31.1 | 77.3 | 56.0 |
|  | *Sebastes entomelas* | 26.2 | 36.2 | 0.0 | 16.7 |
|  | *Sebastes hopkinsi* | 15.9 | 12.9 | 19.4 | 16.4 |
|  | *Sebastes zacentrus* | 10.7 | 8.3 | 0.0 | 3.8 |
|  | *Sebastes paucispinis* | 0.7 | 1.2 | 1.8 | 1.5 |
|  | *Oxylebius pictus* | 4.4 | 2.4 | 0.2 | 1.2 |
| **Hermosa** | *Sebastes spp.* | 38.4 | 40.3 | 96.4 | 68.6 |
|  | *Sebastes entomelas* | 21.3 | 31.7 | 0.6 | 16.0 |
|  | *Sebastes mystinus* | 21.6 | 18.4 | 0.0 | 9.1 |
|  | *Sebastes paucispinis* | 0.5 | 1.0 | 1.6 | 1.3 |
|  | *Oxylebius pictus* | 4.7 | 1.8 | 0.4 | 1.1 |
| **Holly** | *Sebastes hopkinsi* | 18.5 | 16.6 | 51.1 | 28.2 |
|  | *Sebastes entomelas* | 23.6 | 41.5 | 0.0 | 27.6 |
|  | *Chromis punctipinnis* | 7.2 | 4.2 | 14.9 | 7.8 |
|  | *Sebastes caurinus* | 6.3 | 6.1 | 8.5 | 6.9 |
|  | *Sebastes spp.* | 2.9 | 2.9 | 12.6 | 6.1 |
|  | *Sebastes atrovirens* | 9.0 | 5.4 | 0.0 | 3.6 |
|  | *Sebastes paucispinis* | 0.7 | 1.5 | 5.6 | 2.8 |
|  | *Sebastes serranoides* | 1.6 | 3.1 | 1.3 | 2.5 |
|  | *Oxylebius pictus* | 3.6 | 2.7 | 0.9 | 2.1 |
|  | *Ophiodon elongatus* | 0.7 | 1.6 | 3.0 | 2.1 |
|  | *Sebastes auriculatus* | 2.4 | 2.4 | 0.0 | 1.6 |
|  | *Sebastes miniatus* | 3.3 | 2.1 | 0.0 | 1.4 |
|  | *Scorpaenichthys marmoratus* | 0.7 | 1.4 | 0.5 | 1.1 |
| **B** | *Sebastes paucispinis* | 8.2 | 18.6 | 78.8 | 36.4 |
|  | *Sebastes entomelas* | 29.9 | 40.9 | 0.0 | 28.8 |
|  | *Sebastes serranoides* | 6.8 | 11.3 | 3.0 | 8.8 |
|  | *Sebastes mystinus* | 6.4 | 7.3 | 0.0 | 5.1 |
|  | *Chromis punctipinnis* | 14.0 | 5.1 | 0.1 | 3.6 |
|  | *Ophiodon elongatus* | 0.7 | 1.6 | 5.1 | 2.7 |
|  | *Paralabrax clathratus* | 3.6 | 3.0 | 0.0 | 2.1 |
|  | *Sebastes spp.* | 1.7 | 0.8 | 5.2 | 2.1 |
|  | *Sebastes atrovirens* | 5.2 | 2.4 | 0.1 | 1.7 |
|  | *Oxyjulis californica* | 0.3 | 0.3 | 2.9 | 1.1 |
|  | *Sebastes melanops* | 0.2 | 0.5 | 2.3 | 1.0 |
| **A** | *Sebastes mystinus* | 19.7 | 30.5 | 0.0 | 26.4 |
|  | *Sebastes serranoides* | 9.3 | 21.0 | 4.7 | 18.8 |
|  | *Chromis punctipinnis* | 20.6 | 16.0 | 1.4 | 14.1 |
|  | *Sebastes hopkinsi* | 1.0 | 2.0 | 37.2 | 6.7 |
|  | *Phanerodon atripes* | 10.0 | 6.0 | 0.0 | 5.2 |
|  | *Sebastes spp.* | 0.7 | 1.2 | 21.0 | 3.8 |
|  | *Sebastes jordani* | 0.5 | 1.5 | 18.5 | 3.7 |
|  | *Embiotocidae* | 5.1 | 3.9 | 0.2 | 3.4 |
|  | *Sebastes entomelas* | 1.5 | 3.5 | 0.0 | 3.1 |
|  | *Sebastes paucispinis* | 0.4 | 0.7 | 14.5 | 2.5 |
|  | *Sebastes auriculatus* | 1.7 | 2.2 | 0.0 | 1.9 |
|  | *Sebastes atrovirens* | 3.2 | 2.1 | 0.0 | 1.8 |
|  | *Phanerodon furcatus* | 1.9 | 1.4 | 1.3 | 1.4 |
|  | *Rhacochilus vacca* | 6.0 | 1.5 | 0.0 | 1.3 |
|  | *Paralabrax clathratus* | 0.9 | 1.3 | 0.0 | 1.1 |
|  | *Semicossyphus pulcher* | 13.5 | 1.2 | 0.0 | 1.1 |
| **Hillhouse** | *Sebastes paucispinis* | 39.5 | 63.6 | 83.4 | 75.0 |
|  | *Sebastes spp.* | 4.1 | 3.6 | 8.0 | 6.1 |
|  | *Chromis punctipinnis* | 6.1 | 3.4 | 5.8 | 4.8 |
|  | *Sebastes entomelas* | 6.6 | 8.2 | 0.0 | 3.5 |
|  | *Sebastes serranoides* | 7.7 | 8.0 | 0.0 | 3.4 |
|  | *Embiotocidae* | 19.7 | 4.7 | 0.1 | 2.0 |
|  | *Sebastes hopkinsi* | 1.2 | 1.2 | 2.4 | 1.9 |
|  | *Sebastes mystinus* | 5.3 | 3.9 | 0.0 | 1.7 |
| **Habitat** | *Sebastes entomelas* | 58.3 | 73.5 | 0.0 | 61.3 |
|  | *Sebastes hopkinsi* | 24.3 | 11.6 | 46.0 | 17.3 |
|  | *Sebastes paucispinis* | 3.0 | 4.8 | 38.0 | 10.3 |
|  | *Sebastes mystinus* | 3.7 | 2.8 | 0.0 | 2.3 |
|  | *Sebastes spp.* | 1.1 | 0.8 | 7.7 | 2.0 |
|  | *Sebastes serranoides* | 1.3 | 1.8 | 1.0 | 1.7 |
|  | *Chromis punctipinnis* | 1.3 | 0.6 | 3.5 | 1.1 |
| **Gilda** | *Sebastes jordani* | 34.3 | 43.5 | 44.9 | 44.2 |
|  | *Sebastes paucispinis* | 8.7 | 14.6 | 26.6 | 20.5 |
|  | *Ophiodon elongatus* | 3.2 | 5.1 | 9.7 | 7.4 |
|  | *Sebastes spp.* | 2.9 | 2.6 | 7.1 | 4.8 |
|  | *Sebastes entomelas* | 6.6 | 8.4 | 0.0 | 4.3 |
|  | *Scorpaenichthys marmoratus* | 3.9 | 4.3 | 2.8 | 3.6 |
|  | *Sebastes miniatus* | 9.8 | 5.8 | 0.0 | 3.0 |
|  | *Phanerodon atripes* | 1.6 | 1.1 | 4.6 | 2.8 |
|  | *Sebastes semicinctus* | 5.0 | 4.5 | 0.0 | 2.3 |
|  | *Sebastes hopkinsi* | 0.8 | 0.8 | 2.4 | 1.6 |
|  | *Sebastes caurinus* | 1.6 | 1.2 | 1.0 | 1.1 |
|  | *Oxylebius pictus* | 3.5 | 1.9 | 0.2 | 1.1 |
| **Grace** | *Sebastes entomelas* | 59.8 | 75.4 | 0.2 | 58.8 |
|  | *Sebastes paucispinis* | 8.7 | 13.5 | 60.0 | 23.8 |
|  | *Sebastes spp.* | 4.5 | 3.5 | 23.0 | 7.8 |
|  | *Sebastes hopkinsi* | 4.7 | 2.8 | 15.7 | 5.7 |
|  | *Sebastes mystinus* | 1.6 | 1.8 | 0.0 | 1.4 |
| **Gail** | *Sebastes paucispinis* | 32.5 | 49.9 | 49.4 | 49.6 |
|  | *Sebastes spp.* | 6.7 | 10.1 | 32.7 | 20.6 |
|  | *Sebastes hopkinsi* | 3.5 | 4.8 | 12.9 | 8.6 |
|  | *Medialuna californiensis* | 6.2 | 6.7 | 0.0 | 3.6 |
|  | *Chromis punctipinnis* | 20.0 | 5.4 | 1.5 | 3.6 |
|  | *Ophiodon elongatus* | 7.0 | 5.9 | 0.0 | 3.2 |
|  | *Sebastes entomelas* | 1.8 | 3.7 | 0.2 | 2.1 |
|  | *Sebastes rubrivinctus* | 1.6 | 1.2 | 2.0 | 1.5 |
|  | *Sebastes levis* | 6.7 | 2.8 | 0.0 | 1.5 |
|  | *Sebastes rosenblatti* | 4.4 | 2.5 | 0.0 | 1.3 |
|  | *Sebastes macdonaldi* | 3.0 | 2.2 | 0.0 | 1.2 |
| **Edith** | *Chromis punctipinnis* | 53.8 | 44.1 | 83.4 | 63.9 |
|  | *Semicossyphus pulcher* | 16.4 | 14.8 | 0.0 | 7.3 |
|  | *Sebastes jordani* | 0.8 | 4.0 | 7.3 | 5.7 |
|  | *Sebastes hopkinsi* | 0.6 | 2.1 | 5.2 | 3.6 |
|  | *Hypsypops rubicundus* | 6.4 | 6.3 | 0.6 | 3.4 |
|  | *Medialuna californiensis* | 3.1 | 6.0 | 0.0 | 3.0 |
|  | *Scorpaenichthys marmoratus* | 2.9 | 5.0 | 0.2 | 2.6 |
|  | *Girella nigricans* | 2.8 | 5.1 | 0.0 | 2.5 |
|  | *Sebastes spp.* | 0.3 | 0.9 | 2.6 | 1.8 |
|  | *Sebastes semicinctus* | 4.9 | 2.9 | 0.3 | 1.6 |
|  | *Scorpaena guttata* | 1.7 | 2.9 | 0.0 | 1.4 |
| **Elly** | *Sebastes hopkinsi* | 68.7 | 69.7 | 76.5 | 72.9 |
|  | *Sebastes spp.* | 1.7 | 4.6 | 14.0 | 9.0 |
|  | *Chromis punctipinnis* | 1.6 | 2.3 | 7.5 | 4.7 |
|  | *Sebastes entomelas* | 1.6 | 4.9 | 0.0 | 2.6 |
|  | *Scorpaenichthys marmoratus* | 6.4 | 4.7 | 0.0 | 2.5 |
|  | *Ophiodon elongatus* | 4.2 | 3.5 | 0.0 | 1.8 |
|  | *Sebastes paucispinis* | 0.5 | 1.3 | 1.6 | 1.5 |
|  | *Sebastes semicinctus* | 2.7 | 2.0 | 0.0 | 1.0 |
|  | *Medialuna californiensis* | 1.8 | 1.9 | 0.0 | 1.0 |
| **Ellen** | *Sebastes hopkinsi* | 50.5 | 43.4 | 77.8 | 55.9 |
|  | *Sebastes entomelas* | 25.1 | 45.3 | 0.7 | 29.2 |
|  | *Chromis punctipinnis* | 4.1 | 2.3 | 10.1 | 5.1 |
|  | *Sebastes spp.* | 0.9 | 1.4 | 6.7 | 3.3 |
|  | *Sebastes paucispinis* | 0.6 | 1.4 | 4.6 | 2.6 |
|  | *Scorpaenichthys marmoratus* | 7.7 | 2.0 | 0.0 | 1.2 |
| **Eureka** | *Sebastes hopkinsi* | 39.7 | 48.0 | 76.5 | 62.0 |
|  | *Sebastes entomelas* | 22.0 | 17.6 | 0.0 | 8.9 |
|  | *Sebastes spp.* | 3.1 | 3.8 | 9.8 | 6.8 |
|  | *Chromis punctipinnis* | 4.4 | 5.5 | 4.5 | 5.0 |
|  | *Sebastes rufus* | 3.7 | 4.3 | 5.3 | 4.8 |
|  | *Sebastes mystinus* | 5.0 | 5.7 | 0.0 | 2.9 |
|  | *Sebastes paucispinis* | 1.7 | 2.9 | 2.0 | 2.5 |
|  | *Sebastes ovalis* | 4.8 | 3.4 | 1.0 | 2.2 |
