# Supplementary material for: Impacts from Partial Removal of Decommissioned Oil and Gas Platforms on Fish Biomass and Production on the Remaining Platform Structure and Surrounding Shell Mounds
Source: PLoS One. 2015 Sep 2;10(9):e0135812. doi: 10.1371/journal.pone.0135812 (PMC4557934; doi:10.1371/journal.pone.0135812)
Supplement: S3 Table — These values are calculated by dividing the overall values for an entire platform (S1 Table) by the surface area of seafloor beneath the footprint of the platform (Table 1). Standard errors are in parentheses. (DOCX) [file pone.0135812.s003.docx]

|  | **SSB Density (g/m2)** | | | **Somatic Production (g/m2/yr)** | | | **Recruitment Production (g/m2/yr)** | | | **Total Production (g/m2/yr)** | | |
| --- | --- | --- | --- | --- | --- | --- | --- | --- | --- | --- | --- | --- |
| **Platform** | **C** | **PR** | **%** | **C** | **PR** | **%** | **C** | **PR** | **%** | **C** | **PR** | **%** |
| **Irene** | 580.2 | 546.7 | 89.8 | 213.8 | 209.6 | 93.9 | 174.7 | 173.4 | 94.8 | 388.5 | 383 | 95 |
|  | (115.4) | (115.4) | (2.6) | (54.7) | (54.7) | (2.5) | (90.2) | (90.2) | (1.8) | (129.4) | (129.4) | (2.2) |
| **Hidalgo** | 349.6 | 294.9 | 80.1 | 91 | 84.2 | 87.7 | 179.7 | 177.5 | 96.1 | 270.7 | 261.8 | 92.3 |
|  | (64) | (64) | (3) | (21.6) | (21.6) | (3) | (66) | (66) | (1.4) | (86.7) | (86.7) | (2.3) |
| **Harvest** | 285.2 | 268.8 | 90.1 | 77.9 | 75.8 | 95.1 | 89.9 | 89.3 | 94.2 | 167.8 | 165.1 | 95.1 |
|  | (103.5) | (103.7) | (3.1) | (33.2) | (33.2) | (1.4) | (80.4) | (80.3) | (1.8) | (112.7) | (112.6) | (1.5) |
| **Hermosa** | 557.6 | 528.5 | 91.8 | 150.8 | 147.2 | 95.5 | 151.5 | 150.4 | 94.3 | 302.3 | 297.6 | 95.7 |
|  | (143.9) | (143.9) | (2.9) | (44.6) | (44.6) | (1.9) | (112.8) | (112.8) | (2.5) | (149.7) | (149.7) | (1.8) |
| **Holly** | 432.8 | 348.3 | 80.2 | 104.7 | 86.5 | 81.7 | 53.4 | 49.9 | 83.7 | 158.1 | 136.3 | 83.5 |
|  | (48.5) | (42.4) | (2.3) | (20.4) | (17.8) | (2.7) | (20.4) | (20.4) | (4.7) | (34.8) | (32.4) | (2.9) |
| **B** | 412.4 | 330.3 | 83.3 | 93.5 | 85.9 | 90.1 | 35.6 | 35.5 | 99.3 | 129.1 | 121.5 | 91.4 |
|  | (143.9) | (118.9) | (8.6) | (39.5) | (38.5) | (6.9) | (28.7) | (28.7) | (0.6) | (57.3) | (57.3) | (6.2) |
| **A** | 627.9 | 423.8 | 64.4 | 109.9 | 90.7 | 80.9 | 16.8 | 16.6 | 96.8 | 126.7 | 107.3 | 84.7 |
|  | (107.8) | (95.3) | (4.3) | (30.8) | (27.1) | (3.1) | (7.1) | (7.2) | (2.3) | (28.9) | (24.8) | (2.2) |
| **Hillhouse** | 506.2 | 424.6 | 78 | 159.5 | 151.6 | 83.1 | 198.5 | 186.4 | 83.1 | 358 | 338 | 81 |
|  | (245.3) | (249) | (7.9) | (114.9) | (116.3) | (7.9) | (175.5) | (177) | (12.9) | (289.9) | (293.1) | (10.5) |
| **Habitat** | 624.9 | 613.4 | 97.9 | 197.5 | 195.3 | 98.5 | 38.9 | 37.7 | 98 | 236.4 | 233 | 98.5 |
|  | (235.3) | (230) | (0.6) | (89.3) | (88.3) | (0.5) | (16.3) | (15.8) | (1.3) | (100.2) | (98.3) | (0.4) |
| **Gilda** | 287 | 244.5 | 82.5 | 95.7 | 88.7 | 90.5 | 92.5 | 87.7 | 93.7 | 188.2 | 176.4 | 92.5 |
|  | (70.9) | (65) | (3.9) | (25.9) | (25) | (2.6) | (33.3) | (32.3) | (2.9) | (55.4) | (53.5) | (2.5) |
| **Grace** | 1319.9 | 1206.1 | 82 | 416.9 | 414.6 | 97.2 | 118.6 | 118.2 | 94.9 | 535.5 | 532.8 | 97.5 |
|  | (309.8) | (310.2) | (4.4) | (118.9) | (118.9) | (1.2) | (41.4) | (41.4) | (3) | (120.1) | (120.1) | (1.2) |
| **Gail** | 278.7 | 210 | 72.8 | 57.2 | 50.7 | 84.5 | 49.3 | 48.8 | 95.6 | 106.6 | 99.4 | 87.9 |
|  | (27.1) | (26.2) | (2.4) | (11.8) | (11.8) | (1.8) | (20.8) | (20.8) | (1) | (32.1) | (32.1) | (1.8) |
| **Edith** | 828.8 | 136.8 | 18.7 | 86.9 | 22.7 | 27.5 | 88.5 | 15 | 23.5 | 175.4 | 37.8 | 20.1 |
|  | (182.9) | (20.2) | (2.1) | (16.2) | (4.7) | (3.3) | (25.2) | (10.2) | (12.8) | (25.2) | (14.2) | (5.2) |
| **Elly** | 1202.1 | 1108.7 | 88.3 | 159.9 | 153.2 | 91.1 | 142.7 | 134.2 | 70.9 | 302.6 | 287.4 | 86 |
|  | (250.5) | (265.7) | (4.4) | (46.5) | (47.3) | (4.2) | (74.9) | (75.1) | (13.9) | (113.1) | (114.8) | (6.7) |
| **Ellen** | 2463.6 | 2374.6 | 92.8 | 575.9 | 567.4 | 95 | 326.9 | 297.7 | 77.9 | 902.7 | 865.1 | 89.9 |
|  | (870.9) | (868) | (2.2) | (279.7) | (278.4) | (2) | (183.3) | (185.6) | (9.7) | (405.3) | (397.7) | (4.8) |
| **Eureka** | 2149.4 | 2027.1 | 93.2 | 354 | 332.4 | 92.6 | 343.4 | 328 | 90.4 | 697.4 | 660.4 | 92.8 |
|  | (410.8) | (415.8) | (1.4) | (64.5) | (67.3) | (1.6) | (113.2) | (115.2) | (3.1) | (172.2) | (176.4) | (1.5) |

**S3 Table. Mean of annual values scaled to per m^2^ of seafloor beneath the platform for complete platforms (C) and partially removed (PR) platforms and the percent retained after partial removal (%).** These values are calculated by dividing the overall values for a complete or partially removed platform (S1 Table) by the surface area of seafloor beneath the footprint of the platform (Table 1). Standard errors are in parentheses.
