# Supplementary material for: Impacts from Partial Removal of Decommissioned Oil and Gas Platforms on Fish Biomass and Production on the Remaining Platform Structure and Surrounding Shell Mounds
Source: PLoS One. 2015 Sep 2;10(9):e0135812. doi: 10.1371/journal.pone.0135812 (PMC4557934; doi:10.1371/journal.pone.0135812)
Supplement: S4 Table — (DOCX) [file pone.0135812.s004.docx]

**S4 Table. Shell mound mean (SE) of annual density values per m^2^ of seafloor.**

| **Platform** | **Biomass Density (g/m^2^)** | **Somatic Production (g/m^2^/yr)** | **Recruitment Production (g/m^2^/yr)** | **Total Production (g/m^2^/yr)** |
| --- | --- | --- | --- | --- |
| **Irene** | 41.15 | 12.31 | 11.70 | 24.01 |
|  | (8.66) | (2.58) | (2.63) | (4.90) |
| **Hidalgo** | 22.37 | 5.91 | 2.45 | 8.36 |
|  | (6.14) | (2.12) | (1.00) | (2.79) |
| **Harvest** | 18.60 | 3.33 | 0.23 | 3.56 |
|  | (2.22) | (0.66) | (0.10) | (0.65) |
| **Hermosa** | 47.88 | 6.50 | 0.45 | 6.95 |
|  | (4.99) | (2.47) | (0.16) | (2.48) |
| **Holly** | 53.02 | 7.59 | 1.85 | 9.44 |
|  | (22.48) | (2.08) | (0.34) | (2.10) |
| **Gilda** | 138.58 | 34.35 | 34.14 | 68.49 |
|  | (84.85) | (18.66) | (29.08) | (45.18) |
| **Grace** | 72.76 | 8.08 | 2.38 | 10.46 |
|  | (17.27) | (1.53) | (1.03) | (2.32) |
| **Gail** | 31.11 | 4.57 | 0.20 | 4.77 |
|  | (7.36) | (0.89) | (0.04) | (0.87) |
| **Edith** | 113.94 | 25.64 | 4.06 | 29.71 |
|  | (52.52) | (11.83) | (1.57) | (12.63) |
| **Elly** | 176.29 | 24.77 | 11.91 | 36.68 |
|  | (28.38) | (6.10) | (8.09) | (13.82) |
| **Ellen** | 99.73 | 13.83 | 2.48 | 16.30 |
|  | (24.68) | (3.86) | (0.79) | (4.37) |
| **Eureka** | 4.93 | 0.73 | 0.10 | 0.83 |
|  | (1.41) | (0.23) | (0.07) | (0.30) |
