# Supplementary material for: Impacts from Partial Removal of Decommissioned Oil and Gas Platforms on Fish Biomass and Production on the Remaining Platform Structure and Surrounding Shell Mounds
Source: PLoS One. 2015 Sep 2;10(9):e0135812. doi: 10.1371/journal.pone.0135812 (PMC4557934; doi:10.1371/journal.pone.0135812)
Supplement: S5 Table — Only taxa that contribute at least 1.0% of the Total Production are included. Taxa are sorted by percent contribution to Total Production. (DOCX) [file pone.0135812.s005.docx]

**S5 Table. Shell mound percent contribution of individual taxa.** Only taxa that contribute at least 1.0% of the Total Production are included. Taxa are sorted by percent contribution to Total Production.

| **Platform** | **Taxon** | **Biomass** | | | **Somatic Production** | **Recruitment Production** | | **Total Production** |
| --- | --- | --- | --- | --- | --- | --- | --- | --- |
| **Irene** | *Ophiodon elongatus* | 44.6 | | | 7.1 | 82.1 | | 68.9 |
|  | *Sebastes caurinus* | 20.5 | | | 12.4 | 6.2 | | 12.2 |
|  | *Sebastes semicinctus* | 9.3 | | | 47.2 | 6.6 | | 7.5 |
|  | *Sebastes miniatus* | 7.0 | | | 5.6 | 0.0 | | 2.3 |
|  | *Citharichthys sordidus* | 1.4 | | | 2.0 | 2.8 | | 2.3 |
|  | *Oxylebius pictus* | 6.3 | | | 9.3 | 0.0 | | 1.1 |
|  | *Sebastes pinniger* | 1.4 | | | 0.6 | 0.0 | | 1.1 |
| **Hidalgo** | *Ophiodon elongatus* | 49.6 | | | 2.3 | 95.3 | | 65.1 |
|  | *Sebastes semicinctus* | 39.9 | | | 87.4 | 0.0 | | 26.7 |
|  | *Sebastes elongatus* | 2.0 | | | 2.3 | 0.0 | | 1.3 |
|  | *Citharichthys sordidus* | 1.5 | | | 0.7 | 0.6 | | 1.2 |
| **Harvest** | *Ophiodon elongatus* | 9.2 | | | 0.9 | 71.3 | | 30.3 |
|  | *Sebastes saxicola* | 37.4 | | | 49.7 | 0.0 | | 20.1 |
|  | *Sebastes elongatus* | 28.0 | | | 15.7 | 0.0 | | 18.4 |
|  | *Sebastes zacentrus* | 5.3 | | | 8.7 | 0.0 | | 10.3 |
|  | *Sebastes chlorostictus* | 6.7 | | | 4.7 | 0.0 | | 7.4 |
|  | *Sebastes semicinctus* | 5.6 | | | 11.4 | 0.0 | | 4.6 |
|  | *Sebastes rosenblatti* | 1.3 | | | 0.9 | 0.0 | | 1.9 |
|  | *Sebastes spp.* | 1.1 | | | 1.0 | 12.7 | | 1.5 |
| **Hermosa** | *Sebastes semicinctus* | 83.7 | | | 90.3 | 0.6 | | 70.9 |
|  | *Ophiodon elongatus* | 0.8 | | | 0.1 | 82.3 | | 8.4 |
|  | *Sebastes jordani* | 5.6 | | | 2.8 | 0.0 | | 6.9 |
|  | *Sebastes chlorostictus* | 1.7 | | | 0.8 | 0.0 | | 2.8 |
|  | *Sebastes saxicola* | 3.1 | | | 3.0 | 0.0 | | 2.7 |
|  | *Zaniolepis frenata* | 1.4 | | | 0.9 | 3.2 | | 2.0 |
|  | *Citharichthys spp.* | 0.8 | | | 0.3 | 2.6 | | 1.6 |
|  | *Sebastes elongatus* | 1.2 | | | 0.5 | 0.0 | | 1.3 |
| **Holly** | *Sebastes caurinus* | 10.6 | | | 8.4 | 28.3 | | 24.8 |
|  | *Ophiodon elongatus* | 4.5 | | | 0.9 | 22.7 | | 18.2 |
|  | *Sebastes miniatus* | 10.2 | | | 9.0 | 0.0 | | 11.5 |
|  | *Rathbunella spp.* | 1.1 | | | 1.2 | 16.3 | | 7.3 |
|  | *Sebastes dallii* | 23.0 | | | 44.0 | 0.3 | | 6.1 |
|  | *Sebastes auriculatus* | 2.7 | | | 1.1 | 0.0 | | 5.3 |
|  | *Citharichthys sordidus* | 0.4 | | | 1.0 | 10.3 | | 3.1 |
|  | *Sebastes pinniger* | 1.2 | | | 0.5 | 0.0 | | 3.1 |
|  | *Hexagrammos decagrammus* | 1.9 | | | 0.6 | 0.0 | | 2.2 |
|  | *Sebastes semicinctus* | 1.9 | | | 7.1 | 1.0 | | 2.1 |
|  | *Sebastes rubrivinctus* | 1.6 | | | 2.3 | 4.6 | | 1.9 |
|  | *Citharichthys spp.* | 0.2 | | | 0.4 | 5.0 | | 1.5 |
|  | *Merluccius productus* | 0.5 | | | 0.2 | 0.0 | | 1.5 |
|  | *Sebastes rosaceus* | 2.4 | | | 3.4 | 0.0 | | 1.5 |
|  | *Sebastes hopkinsi* | 0.6 | | | 3.1 | 3.6 | | 1.3 |
| **Gilda** | *Sebastes paucispinis* | 18.8 | | | 5.8 | 76.0 | | 59.3 |
|  | *Ophiodon elongatus* | 5.9 | | | 0.7 | 22.4 | | 17.6 |
|  | *Sebastes semicinctus* | 60.6 | | | 85.3 | 0.0 | | 15.9 |
|  | *Sebastes miniatus* | 6.0 | | | 3.3 | 0.0 | | 2.9 |
|  | *Sebastes entomelas* | 1.5 | | | 0.4 | 0.0 | | 1.3 |
| **Grace** | *Ophiodon elongatus* | 8.4 | | | 1.0 | 60.6 | | 41.8 |
|  | *Sebastes semicinctus* | 42.2 | | | 64.3 | 0.0 | | 15.0 |
|  | *Sebastes paucispinis* | 1.9 | | | 0.7 | 30.1 | | 12.9 |
|  | *Sebastes miniatus* | 13.0 | | | 3.1 | 0.0 | | 7.9 |
|  | *Citharichthys spp.* | 4.4 | | | 2.1 | 2.3 | | 5.2 |
|  | *Cymatogaster aggregata* | 18.6 | | | 18.0 | 0.0 | | 4.9 |
|  | *Sebastes hopkinsi* | 1.1 | | | 2.0 | 2.8 | | 1.9 |
|  | *Sebastes chlorostictus* | 1.3 | | | 0.7 | 0.0 | | 1.5 |
|  | *Sebastes mystinus* | 1.0 | | | 0.3 | 0.0 | | 1.3 |
|  | *Sebastes spp.* | 0.7 | | | 1.3 | 2.0 | | 1.2 |
| **Gail** | *Ophiodon elongatus* | 41.2 | | | 1.5 | 21.2 | | 27.6 |
|  | *Sebastes elongatus* | 14.6 | | | 30.8 | 0.0 | | 17.1 |
|  | *Merluccius productus* | 4.3 | | | 2.3 | 0.0 | | 10.1 |
|  | *Sebastes rosenblatti* | 7.9 | | | 7.6 | 0.0 | | 10.0 |
|  | *Sebastes paucispinis* | 5.6 | | | 1.1 | 10.1 | | 10.0 |
|  | *Sebastes saxicola* | 6.2 | | | 17.0 | 0.0 | | 3.6 |
|  | *Torpedo californica* | 2.8 | | | 0.1 | 0.0 | | 3.6 |
|  | *Sebastes simulator* | 6.6 | | | 18.5 | 0.0 | | 2.5 |
|  | *Sebastes macdonaldi* | 2.0 | | | 0.2 | 0.0 | | 2.4 |
|  | *Microstomus pacificus* | 0.4 | | | 1.2 | 23.1 | | 1.9 |
|  | *Sebastes chlorostictus* | 0.8 | | | 1.1 | 0.0 | | 1.1 |
| **Edith** | *Scorpaena guttata* | 59.0 | | | 13.8 | 0.0 | | 47.2 |
|  | *Sebastes semicinctus* | 21.5 | | | 54.9 | 21.1 | | 28.9 |
|  | *Sebastes hopkinsi* | 1.6 | | | 12.5 | 45.3 | | 8.6 |
|  | *Sebastes spp.* | 1.0 | | | 4.5 | 26.8 | | 5.0 |
|  | *Scorpaenichthys marmoratus* | 5.5 | | | 0.3 | 0.0 | | 3.3 |
|  | *Ophiodon elongatus* | 1.9 | | | 0.1 | 0.8 | | 3.3 |
|  | *Rhinogobiops nicholsii* | 1.8 | | | 9.9 | 0.0 | | 1.1 |
|  | *Sebastes jordani* | 0.2 | | | 0.5 | 5.2 | | 1.1 |
| **Elly** | *Sebastes hopkinsi* | 10.4 | | | 27.7 | 71.7 | | 35.9 |
|  | *Ophiodon elongatus* | 50.4 | | | 0.9 | 0.5 | | 28.7 |
|  | *Sebastes semicinctus* | 28.9 | | | 58.1 | 6.1 | | 21.2 |
|  | *Sebastes spp.* | 1.0 | | | 6.8 | 14.0 | | 6.2 |
|  | *Sebastes paucispinis* | 0.3 | | | 0.1 | 3.7 | | 1.8 |
|  | *Sebastes jordani* | 0.3 | | | 0.8 | 1.9 | | 1.2 |
| **Ellen** | *Sebastes semicinctus* | | 53.7 | 81.8 | | | 9.0 | 45.7 |
|  | *Ophiodon elongatus* | | 15.7 | 0.3 | | | 7.0 | 15.9 |
|  | *Citharichthys spp.* | | 2.2 | 1.2 | | | 21.7 | 7.5 |
|  | *Sebastes hopkinsi* | | 2.8 | 4.0 | | | 26.8 | 7.1 |
|  | *Sebastes umbrosus* | | 5.5 | 3.2 | | | 0.0 | 4.6 |
|  | *Sebastes spp.* | | 0.6 | 2.6 | | | 17.7 | 3.7 |
|  | *Sebastes miniatus* | | 3.8 | 1.0 | | | 0.0 | 3.3 |
|  | *Rathbunella alleni* | | 0.4 | 0.2 | | | 4.9 | 2.3 |
|  | *Sebastes paucispinis* | | 0.2 | 0.1 | | | 7.0 | 1.6 |
|  | *Sebastes rubrivinctus* | | 0.9 | 0.7 | | | 4.8 | 1.5 |
|  | *Scorpaenichthys marmoratus* | | 2.9 | 0.1 | | | 0.0 | 1.2 |
|  | *Sebastes rosaceus* | | 3.1 | 1.4 | | | 0.0 | 1.1 |
| **Eureka** | *Microstomus pacificus* | | 4.0 | 5.2 | | | 88.6 | 19.0 |
|  | *Sebastes simulator* | | 44.7 | 51.3 | | | 0.0 | 15.8 |
|  | *Sebastes rosenblatti* | | 8.4 | 4.9 | | | 0.0 | 13.5 |
|  | *Sebastes elongatus* | | 9.6 | 9.0 | | | 0.0 | 11.2 |
|  | *Zoarcidae* | | 1.8 | 0.9 | | | 0.0 | 9.6 |
|  | *Scorpaenichthys marmoratus* | | 6.5 | 0.5 | | | 0.0 | 9.6 |
|  | *Zaniolepis frenata* | | 8.1 | 8.2 | | | 0.0 | 8.0 |
|  | *Zaniolepis spp.* | | 2.4 | 3.0 | | | 2.8 | 3.4 |
|  | *Sebastes helvomaculatus* | | 1.9 | 2.2 | | | 0.0 | 2.5 |
|  | *Sebastomus* | | 6.8 | 7.9 | | | 0.0 | 2.5 |
|  | *Sebastes chlorostictus* | | 1.1 | 0.7 | | | 0.0 | 1.5 |
|  | *Stichaeidae spp.* | | 0.2 | 0.3 | | | 8.0 | 1.4 |
