# Supplementary material for: Impacts from Partial Removal of Decommissioned Oil and Gas Platforms on Fish Biomass and Production on the Remaining Platform Structure and Surrounding Shell Mounds
Source: PLoS One. 2015 Sep 2;10(9):e0135812. doi: 10.1371/journal.pone.0135812 (PMC4557934; doi:10.1371/journal.pone.0135812)
Supplement: S6 Table — (DOCX) [file pone.0135812.s006.docx]

**S6 Table. Shell mound mean (SE) of annual overall values, i.e., density values multiplied by the total area of the shell mound for a given platform (Table 2).**

| **Platform** | **Biomass (kg)** | **Somatic Production (kg/yr)** | **Recruitment Production (kg/yr)** | **Total Production (kg/yr)** |
| --- | --- | --- | --- | --- |
| **Irene** | 555 | 166 | 158 | 324 |
|  | (117) | (35) | (35) | (66) |
| **Hermosa** | 31 | 4 | 0 | 4 |
|  | (3) | (2) | (0) | (2) |
| **Gilda** | 2534 | 628 | 624 | 1253 |
|  | (1552) | (341) | (532) | (826) |
| **Grace** | 1655 | 184 | 54 | 238 |
|  | (393) | (35) | (24) | (53) |
| **Gail** | 20 | 3 | 0 | 3 |
|  | (5) | (1) | (0) | (1) |
